# Validation of food store environment secondary data source and the role of neighborhood deprivation in Appalachia, Kentucky

**DOI:** 10.1186/1471-2458-12-688

**Published:** 2012-08-22

**Authors:** Alison A Gustafson, Sarah Lewis, Corey Wilson, Stephanie Jilcott-Pitts

**Affiliations:** 1 Department of Nutrition and Food Science, University of Kentucky, Lexington, KY, 40506, USA; 2 Department of Public Health Greenville, East Carolina University, Brody School of Medicine, Greenville, NC, 27858, USA

## Abstract

**Background:**

Based on the need for better measurement of the retail food environment in rural settings and to examine how deprivation may be unique in rural settings, the aims of this study were: 1) to validate one commercially available data source with direct field observations of food retailers; and 2) to examine the association between modified neighborhood deprivation and the modified retail food environment score (mRFEI).

**Methods:**

Secondary data were obtained from a commercial database, InfoUSA in 2011, on all retail food outlets for each census tract. In 2011, direct observation identifying all listed food retailers was conducted in 14 counties in Kentucky. Sensitivity and positive predictive values (PPV) were compared. Neighborhood deprivation index was derived from American Community Survey data. Multinomial regression was used to examine associations between neighborhood deprivation and the mRFEI score (indicator of retailers selling healthy foods such as low-fat foods and fruits and vegetables relative to retailers selling more energy dense foods).

**Results:**

The sensitivity of the commercial database was high for traditional food retailers (grocery stores, supermarkets, convenience stores), with a range of 0.96-1.00, but lower for non-traditional food retailers; dollar stores (0.20) and Farmer’s Markets (0.50). For traditional food outlets, the PPV for smaller non-chain grocery stores was 38%, and large chain supermarkets was 87%. Compared to those with no stores in their neighborhoods, those with a supercenter [OR 0.50 (95% CI 0.27. 0.97)] or convenience store [OR 0.67 (95% CI 0.51, 0.89)] in their neighborhood have lower odds of living in a low deprivation neighborhood relative to a high deprivation neighborhood.

**Conclusion:**

The secondary commercial database used in this study was insufficient to characterize the rural retail food environment. Our findings suggest that neighborhoods with high neighborhood deprivation are associated with having certain store types that may promote less healthy food options.

## Background

Obesity prevalence differs significantly among U.S. counties, particularly in the rural, Southern Appalachian region of the U.S. [1,2]. In the 14 counties being studied within this manuscript the range of obesity is between 31% and 37%, compared to the national average of 33% in 2010 [3]. While at the same time, the Appalachian region has been marked by geographic isolation [4] which in turn may influence the health disparities experienced by residents relative to those living in more urban settings [4-6]. There is some evidence to suggest that those living in isolation from resources experience worse health outcomes such as certain cancers [7,8], diabetes prevalence and obesity rates [9] relative to those with greater proximity to health care [10], food stores [11], and physical activity resources [12,13]. What causes these vast differences may be attributed to societal influences, such as the neighborhood food environment and resources, or through effect of social selection [14,15], such as individuals who lead a healthy lifestyle may choose to locate in neighborhoods with healthy food outlets. In order to disentangle the effects the environment has on individual choice, there has been increased attention on measuring the community and consumer food environment as a determinant of health [16].

There have been several international [17-20] and U.S. based [21-23] studies examining the validity of secondary data sources examining the retail food environment at the macro level. However, there are few studies examining the validity of data sources currently used to define and measure the rural community food environment [11,21,24,25]. The lack of consistency between methods and data sources suggests that approaches for measuring the macro-level food environment in rural and remote areas may require different techniques relative to studies conducted in urban settings. To date, one study in Chicago found a positive predictive value (PPV) between commercial data sources and direct field observation of 80% [26]. Most recently in rural South Carolina, the positive predictive value (PPV) was 66% [21] between commercial data source and direct field observation. The results from these two studies suggest that commercial data sources may perhaps have greater validity in urban settings relative to rural areas. One potential reason for the difference between rural and urban settings is that in urban settings the rate of store closings is lower than in rural areas, with 1 in 4 stores closing in 2007 in rural areas compared to 1 in 6 in urban settings. Added to this issue is that a population of 3,252 is needed to support a grocery store in 2010, whereas in 2000 the population needed was only 2,843 [27]. In many of these small census tracts the population is not sufficient to support a store and therefore there may be higher rate of store closings which are not captured with a commercial data source.

Parallel to using valid methods to measure the rural community food environment, especially with higher rates of store closings, is the need to spatially measure access to various food outlets in rural areas to understand the deprivation amplification prevalent in rural and disadvantaged areas [28]. Deprivation amplification suggests that individual or household deprivation (for example, low income) is amplified by area level deprivation (for example, lack of affordable nutritious food or facilities for physical activity in the neighborhood) [29]. Although neighborhood deprivation theory is under much debate [10], in terms of the food environment, findings from the UK suggest that those in remote or disadvantaged areas tend to have adequate access to healthy food resources such as supermarkets [28]. Additionally, other studies conducted in Denmark [30], and Australia [31] corroborates findings from the UK. However, most of these studies were conducted in semi-rural or urban environments or in other countries outside the United States [32], whereas in the Appalachia region there are limited food resources overall, which may suggest that neighborhood deprivation is context specific [28].

Several studies have found that in rural areas supermarket availability does not necessarily indicate an abundant resource of healthy, high quality foods [33,34]. Food environment researchers need to move beyond the assumption that having a supermarket is equivalent to a less deprived neighborhood. This assumption suggests that the presence of supermarkets or healthy food outlets supersedes the effect of fast-food restaurants and less healthy food outlets on health outcomes. Research has recently documented that people with access to supermarket do not necessarily consume more fruits and vegetables or a better body mass index [35,36]. These findings highlight the need to also understand the role of individual choice in store type and in food selection within stores beyond just proximity or access to certain stores. Yet, prior studies show that proximity to fast-food restaurants is associated with more meals consumed at these locations [35]. To explain neighborhood deprivation of food resources in rural areas what might be more meaningful is to examine the coverage area of all food resources in rural settings [25,37,38], which may more accurately depict the overabundance of less healthy food items which nullifies the effect of healthy food outlets [32,38].

Based on the need for better measurement of the food environment in rural settings and to examine how deprivation may be unique in remote, rural settings, the aims of this study were the following: 1) to validate commercially available data source with direct field observations of several food outlet types and 2) to examine the association between neighborhood deprivation and retail food environment.

## Methods

### Study region

The spatial area under analysis consisted of 14 counties in the Appalachia region with a total population of 345,000 people [39]. The study was reviewed and determined exempt from Internal Review Board, as it was secondary data analyses not involving human subjects.

### Census tracts characteristics

Outlets were categorized within their U.S. census tract and a corresponding level of rurality based on the United States Department of Agriculture rural–urban codes ( http://www.ers.usda.gov/Data/RuralUrbanCommutingAreaCodes/2000/). We conducted analyses in 14 (25% of the 54-county Appalachia region) contiguous counties within the 54-county Kentucky Appalachian Regions ( http://www.arc.gov/counties); Owsley, Jackson, Clay, Lee, Estill, Powell, Lincoln, Pulaski, Garrard, Madison, Robertson, Fleming, Montgomery, and Bath. These counties were selected based on location to each other as well as having a diverse sample of counties with different rural codes, a range of 7–9. Descriptive characteristics of counties are shown in Table 1.

**Table 1:** Descriptive of Census Tracts aggregated at County level in rural Appalachia Kentucky, 2011

**Averaged Census Tracts**	**% of individuals with income below poverty level**	**% of families with female headed households with no husband and children under 18**	**% of households with income under $30,000/yr**	**% of households with public assistance income**	**% of people age 16 or older in civilian labor force unemployed**	**% pop in management**	**% of all persons age 25 or older with less than a HS degree**	**% of households with more than one person per room**	**Neighborhood deprivation score (average)**	**p-value difference between census tracts**
Owsley	16.75	3.60	63.40	33.80	6.90	27.50	28.35	1.50	−1.39	0.110
Lee	34.88	6.10	59.40	36.80	17.60	24.10	39.00	0.00	**1.98**	0.02*
Clay	34.32	5.50	55.00	20.90	22.90	21.90	41.02	2.50	**2.38**	0.001*
Jackson	30.16	7.30	47.60	23.50	13.20	23.50	38.87	3.00	1.33	0.334
Bath	26.40	13.20	43.50	21.40	8.10	29.30	29.17	2.10	−0.24	0.703
Lincoln	18.70	6.80	34.60	15.50	7.27	22.90	31.25	1.30	−0.70	0.221
Estill	28.90	7.60	47.00	21.70	9.27	21.50	34.80	0.40	0.44	0.656
Pulaski	24.73	5.90	40.10	15.30	8.60	27.10	29.93	1.10	−0.27	0.445
Montgomery	20.90	7.10	37.90	16.90	4.70	25.10	26.40	1.40	−1.11	0.104
Rockcastle	31.80	4.60	49.10	26.30	10.60	27.00	34.40	0.80	0.55	0.638
Fleming	21.30	5.70	42.80	22.60	8.10	29.50	26.30	2.40	−0.56	0.506
Powell	25.50	9.40	42.30	24.10	9.00	24.90	26.60	1.60	−0.28	0.590
Breathitt	31.10	7.30	56.80	34.30	11.50	22.10	39.50	0.90	**1.98**	0.018*
Madison	19.60	6.80	31.90	11.90	7.70	30.80	17.40	0.90	**−1.67**	0.003*
*Mean*	26.07429	6.921429	46.52857	23.21429	10.38857	25.51429	31.64214	1.421429	0.17	
*Median*	25.95	6.8	45.25	22.15	8.8	25	30.59	1.35	−0.26	
*Range*	5.1-69.6	0-15.2	12.9-80.1	0-25.3	0-52.7	6.1-46.6	7.3-56.9	0-9.1	−4.07-4.34	

*p ≤ 0.05.

### Identifying food outlets via commercial database

Food outlet addresses were purchased from InfoUSA database in July 2011. In most studies to date secondary data sources have been either purchased from InfoUSA or Dunn & Bradstreet as a means to gather large sets of addresses [40,41]. Addresses were categorized based on North American Industry Classification System (NAICS) codes. The categories reflected supercenters (452990), supermarket/grocery stores (Group 445100), convenience stores (446110), gas stations with food marts (447110), fast-casual restaurants (722212), and fast-food restaurants (722213), respectively. Farmers’ markets and produce stands were identified through the health departments’ listing of such vendors. Farmers’ markets were verified through the Kentucky Department of Agriculture. Small grocery stores were categorized based on number of cash registers, less than 5, which has been used as a standard measure for small size stores [16]. Additional criteria for small grocery store was not having a second listing or a known chain within the same county or in another county as used in previous studies [42]. The trained graduate student went into each store to count cash registers as part of the validation efforts described below. Store type was dichotomized has ‘one’ for having any store type and ‘none’ for having zero store type, based on distribution of the data.

### Identifying food outlets via ground-truthing

We conducted ground-truthing [43] to verify the presence of each food outlet in the commercial database and to identify any new outlets (Table 2) in summer and fall of 2011. Ground-truthing is defined as a wind shield audit to verify if the store is located in the same address as InfoUSA has provided and if the location is open. One graduate student was trained in ground-truthing methods and conducted 12 trips, averaging 2 trips per week. Training consisted of both the student and PI driving within the communities with the address to verify location and open status of all stores listed. After one county was jointly performed the graduate student conducted all other assessments. The principal investigator of the study verified addresses by randomly selecting counties and conducted ground-truth verification on 25% of the stores. The field work began in September 2011 and ended in November 2011. Facilities were classified as 1) “**located and open**” (facility was open and found in the database); 2) “**closed**” (outlet listed in database and located but permanently closed); 3) “**not found**” (outlet not found during ground-truthing but was listed in database); or 4) “**ineligible**” (outlet located but not was not within definition of NAICS code assigned) [21]. The original list of stores was obtained from InfoUSA. Outlet name, type, address were recorded for new outlets identified which were not in the Info USA database.

**Table 2:** Comparison of ground-truth to secondary data source listing among 14 counties in rural Appalachia Kentucky 2011

**Data Source and Type of Food Outlet**	**Disposition%**				
	**No. of outlets listed**	**Located & Open**	**Located & Closed**	**Not Found**	**No. of outlets found but not listed**
InfoUSA 2011 All Food Outlets	540	**378**	**27**	**135**	**15**
Grocery Store	26% (140/540)	38% (53/140)	37% (10/27)	53% (71/135)	0
Super Market	6% (31/540)	87% (27/31)	0	3% (4/135)	7% (1/15)
Super Center	1% (5/540)	100% (5/5)	0	0	0
Convenience Store	11% (62/540)	56% (35/62)	26% (7/27)	19% (26/135)	0
Gas station with food mart	22% (120/540)	74% (89/120)	26% (7/27)	18% (24/135)	7% (1/15)
Fast-food and Fast-casual restaurants	28% (151/540)	94% (142/151)	7% (2/27)	5% (7/135)	0
Pizza Parlors	5% (27/540)	81% (22/27)	4% (1/27)	4% (5/135)	7% (1/15)
Dollar Stores	<1% (2/540)	100% (2/2)	0	0	53% (8/15)
Farmer's Markets	<1% (2/540)	100% (2/2)	0	0	13% (2/15)

### Neighborhood deprivation

**Figure 1:**
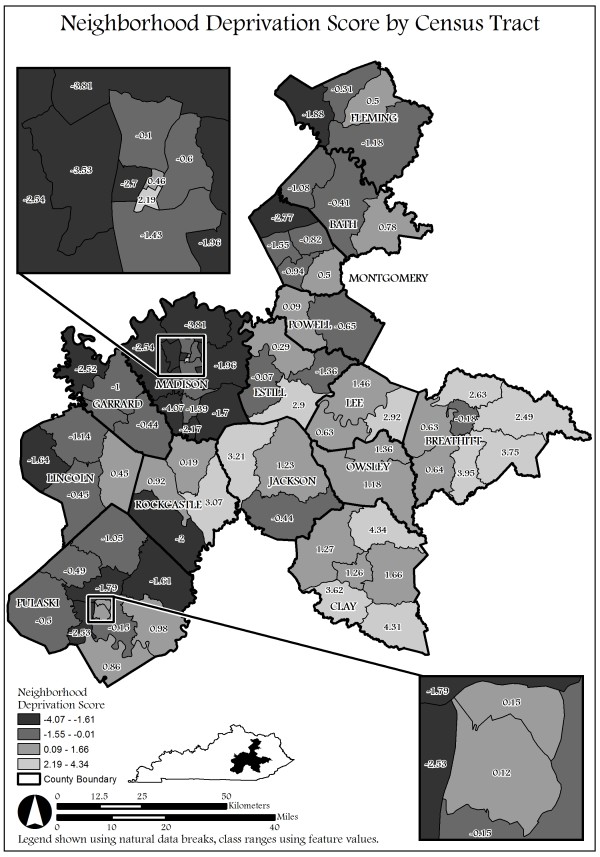
Neighborhood Deprivation Scores in Appalachia, KY.

The Neighborhood Deprivation Index (NDI) was calculated using the method described by Messer et al. [44]. The Index is an empirical score of socioeconomic deprivation based on eight census variables collected from American Community Survey 5-year estimates 2005–2009 [39]: percentage of individuals with income in 2009 below poverty level; percentage of families with female headed households with no husband present and children under age 18 y; percentage of households with incomes under $30,000/year; percentage of households with public assistance income; percentage of people age 16 or older in civilian labor force currently unemployed; percentage of males in management; percentage of all persons age 25 or older with less than a high school degree; and, percentage of households with more than one person per room. We fit a principal component analysis (PCA) to obtain the item loadings, which were used to weight each census variable's contribution to the first principal component. The component was then applied for each census tract within the study area. Neighborhood deprivation retained its linear shape after diagnostic testing of the variable addressing normality. The range of values for NDI was −4.07 – 4.34 (see Table 1 and Figure 1).

### Modified retail food environment index

Coverage represents the number of purchasing opportunities within a given neighborhood [25] or the number of food outlets within a census tract. We calculated a modified retail food environment index [24] ( http://www.cdc.gov/obesity/downloads/NationalActionGuide.pdf) (mRFEI) for each census tract in the Appalachia region. The mRFEI is an indicator of access to retailers that sell healthy foods, like fresh fruits and vegetables. The mRFEI is based on a range from zero (no food retailers that typically sell healthy food) to 100 (only food retailers that sell healthy food).

The mRFEI is constructed for each census tract, by using the following formula:

(1) $$\text{mRFE}=100*\frac{\#\text{Healthy Food Retailers}}{\#\text{Healthy Food Retailers}+\#\text{Less Healthy Food Retailers}}$$

The definitions for healthy and less healthy food retailers are based on the Centers for Disease Control and Prevention definition [45]. Healthy food retailers consist of: grocery stores, supermarkets, supercenters, and produce vendors (produce stores and farmers markets). Less healthy food retailers consist of: fast-food restaurants, gas stations with food marts, and convenience stores, and dollar stores. To date the mRFEI is an environmental indicator of food access or the proportion of healthy stores within a defined neighborhood relative to all the stores accessible [46]. It is not however an indicator of availability of healthy food within the store or availability of unhealthy food items.

Due to the high frequency of food shopping conducted at dollar stores among rural residents [47] but the lack of fresh produce options within this type of store [48], dollar stores were included in the denominator. Dollar stores were tested in the numerator and denominator but results did not significantly change and therefore dollar stores were retained in the denominator. The mRFEI was split into 3 categories based on the distribution of the data (category one = 0; category two = 1–27; category three = 28–100). We conducted sensitivity test for various cut-points and retained high, medium, and low categories based on the results.

### Validation of food outlets in the commercial data base

To characterize the validity of the commercial food venue address database against the ground-truthing field observations, we conducted a sensitivity analysis [21]. The sensitivity analyses consisted of calculating the fraction of food outlets that were listed and found to be open (i.e., “located and open”/(“located and open” + “found, not listed”)). The positive predictive value (PPV) was calculated as the fraction of all listed food outlets that were “located and open” during the field census (i.e., “located and open”/(“located and open” + “closed but listed in database” + “not found during ground-truthing but listed in database”)). The final categories consisted of 1) located and open; 2) located and closed; 3) not found; 4) not listed but found. Because of structural zeroes, chance-adjusted kappa statistics could not be computed. We calculated an exact binomial confidence interval for each proportion. Fisher’s exact tests were used to evaluate accuracy due to small cell size (Table 3).

**Table 3:** Validity of secondary data source as compared to ground-truth effort among 14 counties in rural Appalachia Kentucky 2011

**Data Source and Type of Food Outlet**	**Sensitivity**	**95% CI**	**PPV**	**95% CI**
**InfoUSA 2011**				
All Food Outlets	0.96	0.89, 1.03	0.7	0.67,0.73
Grocery Store	1.00	1.00, 1.00	0.38	0.32, 0.45
Super Market	0.96	0.85, 1.07	0.87	0.83, 0.95
Super Center	1.00	1.00, 1.00	1	1.00, 1.00
Convenience Store	1.00	1.00, 1.00	0.56	0.48, 0.60
Gas station with food mart	0.99	0.85, 1.08	0.74	0.69, 0.87
Fast-food and Fast-casual restaurants	1.00	1.00 , 1.00	0.95	0.89, 1.02
Pizza Parlors	0.96	0.82, 1.09	0.81	0.75, 0.89
Dollar Stores	0.20	0.14, 0.24	1	1.00, 1.00
Farmer's Markets	0.50	0.41, 0.62	1	1.00, 1.00

Abbreviations: CI, confidence interval; PPV, positive predictive value.

The sensitivity percentage can be interpreted as the ability of the InfoUSA data base to accurately capture the food outlets that are listed. A sensitivity of 100% is deemed to be highly sensitive, while 50- 70% is moderate, and less than 50% is low [21]. The PPV can be interpreted as the likelihood that an establishment is open and found. We used cut-points of below 0.30 as poor, 0.31-0.50 as fair, 0.51-0.70 as moderate, from 0.71-.90 as good, and over 0.91 as excellent [18,49].

## Statistical and geospatial analysis

All analyses were conducted using Stata 11.0 version [50]. To test for differences between census tracts within counties on neighborhood deprivation scores, t-tests were used with a Type I error rate of 0.05. The hypothesis originally proposed asked whether neighborhoods with more deprivation would have less healthy stores or a lower mRFEI. To test this hypothesis multinomial logistic regression was used to model the association between neighborhood deprivation and mRFEI. Our secondary hypothesis asked whether neighborhoods with a specific type of store would have more or less deprivation. To test neighborhood deprivation for each store type (super center, super market) logistic regression was used. A measurement error correction factor was added for all models due to the sensitivity and positive predictive value results [51] and to improve retail food environment estimates. The measurement error correction was set at .3 using C + simex commands. We used census tracts with zero for store values as the reference group for models testing the association between neighborhood deprivation and mRFEI. We used no store in census tract relative to having a store for models testing the association between neighborhood deprivation and each store type (e.g. Super Center). Additionally, research thus far has used zero as the referent to look at neighborhood deprivation in food deserts relative to neighborhoods with adequate variability of store types [52].

## Results

Figure 1 depicts the spatial distribution of the census tracts within the counties for each level of neighborhood deprivation. For ease of graphical representation various levels of deprivation have been shown. The two extreme levels of deprivation are indicated by light gray and dark gray. Low neighborhood deprivation is depicted by light gray with a range in scores of −4.07 - -1.51 while high neighborhood deprivation is depicted by dark gray with a range in scores of 2.19 – 4.34. Figure 1 graphically indicates there are many census tracts with high deprivation clustered together within counties. Additionally several census tracts with high deprivation are next to census tracts in other counties with high deprivation.

**Figure 2:**
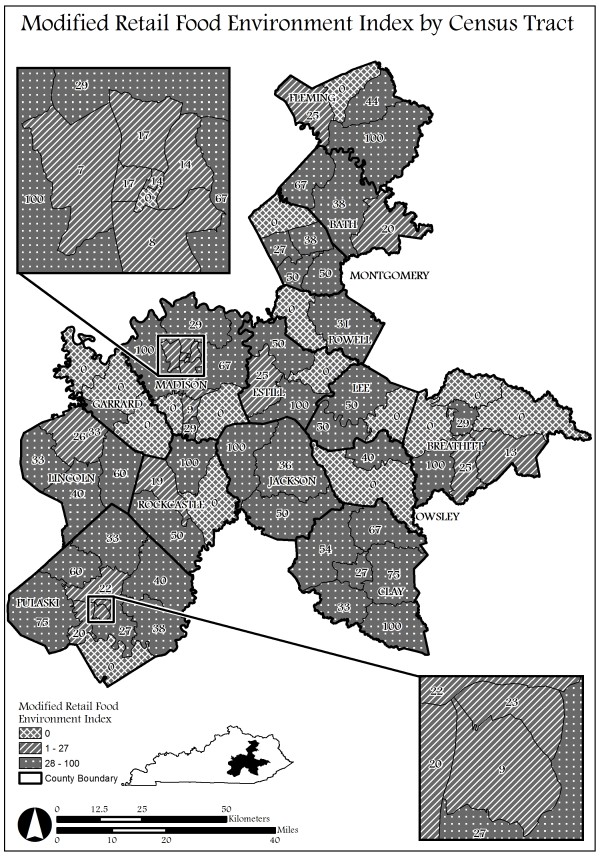
Retail Food Environment Index Scores in Appalachia, KY.

Figure 2 depicts the spatial distribution of the census tracts within the counties for each level of the modified retail food environment index (mRFEI). Each level of the mRFEI is shown by shades of gray and with line or dot mark patterns. The census tracts that are shaded light gray with cross hatch marks indicate no stores or zero. The census tracts with a mRFEI score of 1–27 that have diagonal lines indicate a low ratio of healthy stores relative to all stores within the census tract. The census tracts with a mRFEI score of 28–100 that are dark gray with dots indicate a high ratio of healthy stores relative to all stores within the census tract. Similar to the neighborhood deprivation clustering pattern, those census tracts with no stores tend to cluster within the same county. However, graphically there are different patterns between counties that have a low mRFEI adjacent to census tracts with high mRFEI. While some counties have all census tracts with high mRFEI scores, other counties have one census tract with low mRFEI scores next to census tracts with high mRFEI scores.

Table 1 shows the descriptive statistics for each county in the rural Appalachian area of the variables the are used to create the neighborhood deprivation score. Most of the counties experience high rates of poverty and unemployment. The mean percentage of poverty among all counties is 26.07% with a range of 5.1-69.6%. The mean percentage of unemployment among those 16 years of age and older is 10.38% with a range of 0–52.7%. The mean neighborhood deprivation score for all counties was 0.17 with a range of −4.07 – 4.34. There are also significant differences between census tracts within counties for neighborhood deprivation scores but in fewer counties; 4 of the 14.

Table 2 compares findings from direct observation (ground-truthing) to the secondary commercial database for all stores and for each store type. Of all the stores found in the commercial database, (n = 540), there were a total of 378 open and located (70%), 27 located and closed (5%), and 135 not found (25%). Additionally, there were 15 stores not on the commercial database list but that were located. Of the 540 stores on the original InfoUSA list, the most common type of stores are fast-food and fast-casual restaurants (28%, 151/540), and grocery stores (26%, 140/540). The type of food outlet with the greatest likelihood of being on the original list and located and open was supercenters (100%), followed by fast-food and fast-casual restaurants (94%, 142/151). The type of food outlet with the lowest likelihood of being on the list and located and open was small non-chain grocery stores (38% 53/140). Additionally, the lowest percentage of stores not listed and found was dollar stores (53% 8/15).

Table 3 highlights the validation results of the commercial database versus direct observation (ground-truthing) (% agreement, sensitivity, PPV). Results indicate that the sensitivity of the commercial database was very high for traditional food outlets (grocery stores, supermarkets, convenience stores), with a range of 0.96-1.00. These results indicate that InfoUSA commercial database is highly sensitive for traditional food retailers overall. If a traditional store type was listed and open close to 100% of the time InfoUSA listed this store on their list of addresses. However, the sensitivity for non-traditional food outlets was low compared to traditional food venues with a range of 0.2-0.5. These results indicate the InfoUSA commercial database is not sensitive to non-traditional food venues.

Specifically, the sensitivity result for supermarkets, a traditional food venue, was 0.96. This result indicates that 96% of the time if a store was on the InfoUSA database it was located and open per direct observation. A similar result was found for supercenters; 100% of the time the supercenter was located and open per direct observation relative to the InfoUSA data base. However, the sensitivity was much lower for non-traditional food venues (Dollar stores (0.2) and Farmer’s Markets (0.5)). Dollar stores and Farmer’s Markets were found through the ground-truthing approach but were not listed on the InfoUSA commercial list.

Similar to the sensitivity analyses the PPV was excellent for supercenters with a PPV of 1.0. The PPV for super markets was a bit lower with a PPV of 0.87 indicating the InfoUSA was good compared to direct observation. However, the PPV was much lower for small grocery stores, with a score of 0.38 indicating InfoUSA was a poor measure for assessing if stores are actually open compared to direct observation. There were a low percentage of stores open when they were located through direct observation. Although the store was found, a small percentage of the stores were actually open; only 38%. Lastly, the PPV was high for dollar stores and Farmer’s Markets, at 100% was excellent, as we found stores listed on the commercial database 100% of the time.

Table 4 shows the results of the association between neighborhood deprivation and the mRFEI. There was no association between neighborhood deprivation and the mRFEI. However, when stratified by store type the results indicate that neighborhoods with low deprivation have lower odds of having at least one super center [OR 0.50 (95% CI 0.27. 0.97)] and convenience store [OR 0.67 (95% CI 0.51, 0.89)] compared to those with no store types and higher deprivation. Such that, neighborhoods with low percentages of poverty, unemployment, and other economic indicators have a low probability of having super centers and convenience stores in their neighborhood compared to those with high deprivation Additionally, we did not find that neighborhoods with low deprivation have more grocery stores or super markets.

**Table 4:** Neighborhood Deprivation and the association with Modified Retail Food Environment Index and Stratified Store Type, Appalachia Kentucky, 2011

	**Neighborhood Deprivation Score (Z-score)**	**p-value**	**95% CI**
mRFEI Score*			
Low mRFEI Score (0 no stores)	−1.3	0.19	−0.7, 0.11
Medium mRFEI Score (1–27)	−1.9	0.06	−0.71, 0.01
High mRFEI Score □ (28–100)	REF	REF	REF
**Stratified Store Typeδ**	**Neighborhood Deprivation Score (Odds Ratio)**	**p-value**	**95% CI**
Super Center (1 or more)	0.51	**0.04**	0.27, 0.97
Grocery and Super market (1 or more)	0.99	0.95	0.75, 1.31
Gas stations (1 or more)	0.87	0.15	0.62, 1.08
Convenience Stores (1 or more)	0.67	**0.01**	0.51, 0.89
Fast-Food Restaurants including take-out pizza (1 or more)	0.81	0.11	0.64, 1.05
Dollar Stores (1 or more)	0.95	0.87	0.72, 1.52

* Modified Retail Food Environment Index mRFEI = (Healthy Food Outlets)/(Healthy Food Outlets + Less Healthy Food Outlets) *100.

□ Reference is High mRFEI.

δ Reference is ‘none’ for each store type.

## Discussion

Research regarding the role of the macro-level food environment has experienced a surge in publications in recent years [53,54], with many studies using secondary data sources as a way to classify neighborhoods with regard to access and availability of food stores [41,55,56]. Reliance upon secondary data sources has led to substantial measurement error [17,21,48]. Our findings provide further evidence to support conducting direct observation or ground-truthing in rural settings to verify the presence of food venues in the retail food environment [21,48] obtained from commercial data sources. Previous studies assessing the macro-level food environment, such as number and type of food outlets in a neighborhood, may have introduced bias by not conducting validation studies. This may explain why results of such studies examining association and between the retail food environment and neighborhood characteristics have been conflicting [34,40,57-59].

To date, there are few studies using several approaches to characterize the macro-level food environment, with fewer studies reporting on validation efforts [21,43] among rural settings. Our results are similar to a previous study conducted in some rural locations in South Carolina [21] such that there were low positive predictive values for non-traditional food outlets, such as dollar stores and pharmacies. Our secondary data source had greater sensitivity which may be due to the separation of grocery stores from supermarkets, geographic difference between the studies, and the high percentage of establishments not being found in the South Carolina study. The South Carolina study found many establishments, yet they were closed. Additionally, the previous study validated locational presence from several secondary data sources, whereas in this study we only validated one secondary data source with direct observation. However, we conducted our analyses in 14 rural counties to specifically address accuracy of food venues in rural areas. As previous research has shown, rural residents shop for food in non-traditional food outlets such as dollar stores, farmer’s markets, and gas stations with marts [47,60]. Relying solely on one secondary data source to determine location of key establishments would introduce a biased measure of the retail food environment. Future studies should consider employing direct observation for measuring the retail food store environment, especially in rural areas.

We did not find an association between neighborhood deprivation and the retail food environment for census tracts with no retail stores. This is consistent with several studies, both in the U.S. [61,62] and internationally [32,63]. However, in our study those neighborhoods with lower deprivation were less likely to have a super center or a convenience store in their neighborhood. This result is consistent with studies conducted in rural settings [11,28]. The dynamic between deprivation and the retail food environment is complex. Given that neighborhoods with high deprivation generally have less population, and lower income per individual, there is less incentive for chain food outlets to open stores [64]. With less opportunities to purchase food individuals in remote areas and with more deprivation face greater odds of having access to stores in general [11] and especially stores that sell affordable healthy options [24,34]. Added to this dynamic is the difficulty that individuals in remote areas face with regard to travel time to certain locations [28]. Taken together, there are limited opportunities for economic development in these areas, especially for large supermarkets or grocery stores, which tend to sell the highest percentage of healthy items at the best prices [65]. These findings suggest that the degree of neighborhood deprivation may play a role in access and availability of healthy food options in rural areas [66].

There are several limitations to our study. We do not have data on consumer shopping patterns and behaviors. It is highly likely that residents living in neighborhoods with no stores shop for food in adjacent neighborhoods with retail food stores. The actual food environment individuals are exposed to are adjacent to where they reside [67]. The results did show that several of the census tracts with zero stores are in point of fact adjacent to census tracts with stores (Figure 2). However, in some cases the proportion of stores favored healthy options while in other cases the proportion of stores favored unhealthy options. Suggesting that individuals are able to access food outlets, yet those outlets may or may not have an abundance of healthy items. The mRFEI is a measure of proportion and does provide the context of availability where individuals shop. Therefore a strong limitation to our study is the lack of both consumer food environment measures and macro level measures such as number and type of stores within a neighborhood. Availability of food within the stores may be more relevant in regards to purchasing behaviors and dietary intake [68] which has not been captured in this study. Future research should examine how living in a neighborhood with no retail food outlets influences food purchasing habits and travel patterns over time, while also assessing the consumer food environment within the stores where individuals shop.

Lastly, we only used one source of secondary data and therefore our sensitivity and positive predictive values might have been higher or lower had more secondary data been collected and validated. Previous studies using more than one secondary data source have found lower values overall [21].

Strengths of this study are the rather large effort at conducting ground-truthing across a rural and remote area. Few studies have been able to verify food venue location in a rural remote setting [48]. Additionally, this study has provided further evidence between store type and deprivation in rural areas of the U.S.

## Conclusion

This study provides further support for the need to conduct direct observation of retail food stores when characterizing the food store environment, especially in rural areas, due to the low sensitivity and positive predictive values for certain types of food retailers. This study also suggests that in rural areas, neighborhood deprivation is associated with having certain store types which may or may not sell healthy food items. It is suggested that policies and development aimed at improving healthy food access and availability in rural areas is a promising public health strategy for those most in need.

Figure 1 76 census tract neighborhoods within 14 counties in the Appalachia region of Kentucky. The shaded census tracts represent neighborhood deprivation score for that census tract within the county. The various shades of gray represent 4 different categories of neighborhood deprivation. The most extreme ends of the spectrum for neighborhood deprivation are indicated with dark gray on one end and light gray on the other end. Dark gray is low neighborhood deprivation (i.e. low rates of unemployment; low rates of poverty a range of −4.07–−1.51). Light gray is high deprivation (i.e. high rates of unemployment; high rates of poverty a range of 2.19–4.34).

Figure 2 76 census tract neighborhoods within 14 counties in the Appalachia region of Kentucky. The shaded and patterned census tracts represent the modified retail food environment index score within the county. The census tracts that are shaded light gray with cross hatch marks indicate no stores or zero. The census tracts with a mRFEI score of 1–27 that have diagonal lines indicate a low ratio of healthy stores relative to all stores within the census tract. The census tracts with a mRFEI score of 28–100 that are dark gray with dots indicate a high ratio of healthy stores relative to all stores within the census tract.

## Competing interests

The authors have no conflict of interest to declare.

## Authors’ contributions

SL assisted with data collection. CW assisted with development of the maps. SJ-P assisted with writing and revising of the manuscript. AG conducted data analysis, interpretation of the data, writing, revising, and decision of publication. All authors read and approved the final manuscript.

## Funding

Funding was provided from the University of Kentucky Research Foundation.

## Pre-publication history

The pre-publication history for this paper can be accessed here:

http://www.biomedcentral.com/1471-2458/12/688/prepub
